# Transcriptome analysis reveals hypoxic response key genes and modules as well as adaptive mechanism of crucian carp (*Carassius auratus*) gill under hypoxic stress

**DOI:** 10.3389/fimmu.2025.1543605

**Published:** 2025-02-05

**Authors:** Mengchao Xing, Zhen Rong, Xin Zhao, Xiaowei Gao, Zhiguang Hou, Lihan Zhang, Waiho Khor, Yihuan Xu, Li Chen, Chengbin Wu

**Affiliations:** ^1^ Ocean College, Hebei Agricultural University, Qinhuangdao, China; ^2^ Hebei Key Laboratory of Aquaculture Nutritional Regulation and Disease Control, Hebei Agricultural University, Qinhuangdao, Hebei, China; ^3^ Higher Institution Centre of Excellence (HICoE), Institute of Tropical Aquaculture and Fisheries, Universiti Malaysia Terengganu, Kuala Terengganu, Malaysia; ^4^ Ocean and Fisheries Science Research Institute of Hebei Province, Department of Science and Technology of Hebei Province, Qinhuangdao, China; ^5^ Hebei Key Laboratory of Marine Biological Resources and Environment, Department of Science and Technology of Hebei Province, Qinhuangdao, China

**Keywords:** *Carassius auratus*, hypoxia-tolerant, adaptive mechanism, RNA-Seq, Bayesian networks

## Abstract

Fish gill tissue is a primary organ responsive to acute oxygen deprivation or dissolved oxygen (DO) fluctuations in aquatic environments. However, the adaptive mechanism of crucian carp to hypoxic stress remains largely unknown. Here, we investigated gill physiological and transcriptomic changes of crucian carp exposed to hypoxic conditions (dissolved oxygen concentration of 0.6 ± 0.3 mg/L) for different durations (0 d, 1 d, 2d, 3d, 4 d, and 5d). Transcriptomic analysis revealed that the hypoxia group (0.6 ± 0.3 mg/L DO) exhibited a reduction in interlamellar cell mass (ILCM) on the gill filaments, compared with the control group (6.6 ± 0.3 mg/L DO). With prolonged hypoxia stress, the epithelial cells in the gill lamellae became sparse at 3 d to 5 d, and gill vacuoles were increased. A total of 3,502 differentially expressed genes (DEGs) were identified, and 3 hypoxia-specific modules were screened through differential expression analysis, weighted gene co-expression network analysis (WGCNA), and Bayesian network analysis. The apoptosis, necroptosis, efferocytosis and FoxO signaling pathways were significantly enriched based on the KEGG enrichment pathway analysis. The VEGF pathway genes are significantly expressed, enhancing the generation of microvessels in the gill filaments, and improving the capacity to carry oxygen, thus enabling the crucian carp to adapt to hypoxia stress. Hypoxia activated glycolysis, enhanced anaerobic metabolism, promoted β-oxidation of fatty acids, providing energy and maintaining normal physiological metabolism, eventually improving antioxidant and immune capabilities in crucian carp. In summary, this study reveals the molecular mechanism by which crucian carp adapt to hypoxic stress. Our findings provide valuable references for promoting the healthy aquaculture of hypoxic-sensitive fish and breeding hypoxia-tolerant fish varieties.

## 1 Introduction

Dissolved oxygen (DO) is a crucial ecological factor for fish and other aquatic animals (1). DO in aquaculture water is unstable, and it may be decreased due to multiple factors such as weather variation, pollution, and high-density farming (2). Hypoxia refers to a state in which the DO level is excessively low, thus adversely affecting fish behavior, growth, and physiological metabolism and eliciting immune responses (3). Severe hypoxic stress can pose a significant threat to fish survival (4). With increasingly severe global warming and water pollution, hypoxia has caused enormous economic losses in the aquaculture industry, and thus it is essential to explore the mechanism of hypoxia adaptation for fish breeding (5).

Gill of fish is the principal respiratory organ responsible for gas exchange with the surrounding aquatic environment (6). In a hypoxic or anoxic environment, fish bodies can produce a series of response mechanisms, mainly including increasing the number of red blood cells, enhancing the oxygen carrying capacity of hemoglobin, stimulating angiogenesis, activating the antioxidant defense system, and changing the metabolic mode (7, 8). It has been reported that exposure to hypoxia may result in a series of physiological and morphology of fish gill in multiple fish species (9). For example, blunt snout bream (*Megalobrama amblycephala*) *(* 10), mangrove killifish (*Kryptolebias hermaphroditus*) (2) and hybrid yellow catfish (*Tachysurus fulvidraco* ♀×*Pseudobagrus vachellii* ♂) (1) respond to hypoxia stress by reducing the size of their interlamellar cell masses (ILCMs), exposing the lamellae, and increasing the gill-water contact area. Nilsson et al. (2012) (11) speculated that apoptosis might be an important molecular mechanisms of gill remodeling in fish. Additionally, fish have been found to enhance their anaerobic metabolism to sustain their energy supply in response to DO insufficiency (12). Therefore, some studies have investigated the activity changes in lactate dehydrogenase (LDH), a key enzyme regulating anaerobic metabolism, under hypoxic stress, and found the LDH enzyme activity in gill tissue of hybrid sturgeon (*Acipenser schrenckii* ♂×*Acipenser baerii ♀*) (13) and Hong Kong *oyster Crassostrea hongkongensis *(14) showed an upward trend under hypoxic stress. Moreover, hypoxia can also interfere with the electron transport chain, leading to the over production of reactive oxygen species (ROS) (15). Excessive ROS can induce oxidative damage to cellular membranes, proteins, and DNA, hence resulting in programmed cell death (also known as apoptosis) (16). Superoxide dismutase (SOD) and catalase (CAT), as two important antioxidative enzymes, play a significant role in removing ROS and alleviating the damage caused by oxidative stress in fish. Typically, the concentration of malondialdehyde (MDA) serves as an indicator of oxidative stress in fish (17).

Crucian carp (*Carassius auratus*) are affordable and delicious, exhibiting high quality and high nutrition, meanwhile being excellent source of protein (18). Crucian carp can survive hypoxic aquatic environment for a long period. Exploring the adaptation mechanisms of crucian carp gills to hypoxic environments will be conductive to alleviating the stress of hypoxia in aquaculture. The adaptation of hypoxia-tolerant freshwater turtles (*Trachemys and Chrysemys genera*) to hypoxia has also been extensively studied (19, 20). However, sea turtles clearly adopt a different survival strategy, entering a state similar to deep dormancy when exposed to hypoxia (21).

With the advancement of transcriptome technology and the decrease in next-generation sequencing cost, RNA sequencing (RNA-seq) has emerged as a powerful tool for investigating stress response mechanisms (7). In the pufferfish (*Takifugu rubripes*), as hypoxia persists, lipid metabolism has been found to become the primary energy supply pathway (22). In the darkbarbel Catfish (*Pelteobagrus vachelli*), apoptosis-related genes are upregulated, and antioxidant capacity is enhanced under hypoxia stress (23). However, the molecular mechanisms underlying cell apoptosis in crucian carp gill tissue remain largely unclear.

The goal of this study was to decipher the response mechanism of crucian carp gills to hypoxic stress. First, we conducted RNA-seq analyses and compared gill transcriptional profiles between hypoxia-stressed group and control group. The results demonstrated that under hypoxic stress, the pillar cells in the gill lamellae became sparse, and gill vacuoles were increased, thus enhancing anaerobic metabolism, eventually improving antioxidant and immune capabilities of the gill tissue. Based on differential expression analysis, weighted gene co-expression network analysis (WGCNA), and Bayesian network analysis, a total of 3,502 differentially expressed genes (DEGs) were identified, and 3 hypoxia-specific modules were screened. Further, 11 hypoxia response-related key genes were identified such as *pak1, cdc23, smad3a*, and *caspase7*. Additionally, this study also revealed the effects of hypoxia stress on the activity of antioxidative enzymes SOD and CAT in crucian carp gill tissue. Our findings offer new insights into the molecular mechanisms of hypoxia tolerance in crucian carp and provide valuable reference for breeding hypoxia-tolerant fish varieties.

## 2 Materials and methods

### 2.1 Ethical approval

All the experiments were approved by the Animal Experimentation Ethics Committee of Hebei Agricultural University (Grant No.2022075), following the Laboratory Animal Welfare Guidelines of China (GB/T 35892-2018).

### 2.2 Experimental procedures

All 400 crucian carps were from a pond (in Zhangjiakou City, Hebei Province, China) and were reared in a 22,500-liter water tank for two weeks of acclimation. The fish were not fed 24 hours before the hypoxia experiment. The crucian carp were divided into two groups with three parallel glass tanks (1.0 m × 1.0 m × 1.5 m) per group. For the control group (C), 150 fish (50 fish per tank, with a body weight of 94.5 ± 4.5 g) were randomly selected and reared in a normoxic aquatic environment (with a dissolved oxygen (DO) level of 6.6 ± 0.3 mg/L at 22.5 ± 0.5°C). For the treatment group (T), 150 fish (50 fish per tank, with a body weight of 94.5 ± 4.5 g) were randomly selected and reared in a hypoxic aquatic environment (with a DO level of 0.6 ± 0.3 mg/L at 22.5 ± 0.5°C). As previously described (24, 25), during the entire experiment, a glass cover of the same size was placed above the glass tanks. A 1-cm³ small hole was left in the middle of the glass cover, through which a nitrogen (N₂) inflation tube and an oxygen (O₂) inflation tube passed and were wound under a cubic iron frame. There was an air valve piston on each of the two inflation tubes to control the DO in the experiment. The DO was continuously measured by an oxygen electrode (YSI, ProODO, Germany) (**Supplementary Figure S1**). In this study, the loss of equilibrium (LOE) of the fish in the water was defined as the hypoxic state of the fish (26). During the 5-day hypoxia experiment, samples were taken at five time points (1 day, 2 days, 3 days, 4 days, 5 days). Three fish were taken from each glass tank. At each sampling point, the crucian carp were anesthetized with 0.5 g·L⁻¹ tricaine methanesulfonate (MS-222, Sigma, United States), and the second gill of each crucian carp was collected. A part of the gills was placed in 4% paraformaldehyde (Biosharp, China) for subsequent morphological analysis, a part was placed in a 2-mL cryopreservation tube and sent to a company for transcriptomics analysis (RNA-seq), and the remaining gill tissues were collected into 5-mL EP tubes and frozen at -80°C for subsequent experiments.

### 2.3 Analysis of gill biochemical parameters

The 100 mg gill sample was homogenized in 900 μL pre-cooled phosphate-buffered saline (PBS) solution (pH 7.2) for the enzyme activity determination. The activities of SOD (24), CAT (25), LDH (12) and the oxidative product MDA (25) level were determined using commercial kits by previously reported method (26).

### 2.4 Histological observation

After paraffin sectioning and hematoxylin-eosin (H&E) staining, gill samples were subjected to histological observation, as our previously described (27). Briefly, gill samples were rinsed by PBS solution to remove residual impurity, then immersed in Bouin’s solution, and finally stained with the H&E. These sections were observed under Leica microscope (Wetzlar, Germany).

### 2.5 RNA extraction and transcriptome sequencing

Total RNA was extracted from fish gill tissue (n = 3 per group) using TRIzol reagent (Invitrogen, Carlsbad, CA, USA), and RNA integrity and total quantity were determined using Agilent 2100 bioanalyzer. This analysis was conducted with three replicates per group, so that eighteen samples were prepared in total (C_1, C_2, C_3, T1_1, T1_2, T1_3, T2_1, T2_2, T2_3, T3_1, T3_2, T3_3, T4_1, T4_2, T4_3, T5_1, T5_2, andT5_3).The PCR products were purified using AMPure XP beads to remove primer dimers and other unwanted byproducts, and finally high-quality cDNA library was obtained. Upon completion of library construction, an initial quantification was performed using a Qubit 2.0 Fluorometer to obtain the accurate concentration of the cDNA library. Then, the library was diluted to a working concentration of 1.5 ng/μl. The quality of the cDNA library was assessed using an Agilent 2100 Bioanalyzer to ensure that the library was qualified for downstream sequencing. After quality control, the qualified libraries with valid concentration and required offline data size were pooled, and subjected to the Illumina sequencing to obtain 150 bp paired end reads (28).

### 2.6 Assembly and annotation of transcripts

In raw data pre-processing, strict quality control was implemented to eliminate specific contaminants from sequencer by adapter trimming, to remove ambiguous nucleotides by N-clipping, and to filter the reads with a Qphred cutoff of ≤5 and read length < 50% (29). After quality control, the multiple indicators of the obtained clean reads were assessed, including Q20 and Q30 quantifications for base-calling accuracy, and GC content analysis. The resulting high-fidelity data lay the foundation for downstream analyses. The reference genomes were retrieved, and the transcripts were annotated based on the genome database NCBI (https://www.ncbi.nlm.nih.gov/datasets/genome/?taxon=7957,217509). A reference index was generated with HISAT2 v2.0.5 (30), followed by aligning paired-end reads to this index for precise mapping.

### 2.7 Identification of differentially expressed genes

Relative gene expression levels of transcripts were standardized by fragments per kilobase of exon model per million mapped reads (FPKM) (31). The differential expression (DE) analysis across two biological replicates was performed using the DESeq2 v1.20.0 by applying negative binomial distribution models to determine variability and significance (32). The Benjamini-Hochberg (BH) procedure was utilized for multiple test correction to control the false discovery rate (FDR). The DEGs were identified with the thresholds of |log2 FC (fold changed)| ≥ 1 and adjusted *P*-value<0.01. The Gene Ontology (GO)(http://geneontology.org/) and Kyoto Encyclopedia of Genes and Genomes (KEGG) enrichment analyses were performed to determine the potential functions of the DEGs and their significantly enriched metabolic pathways, and P < 0.05 was considered as significant enrichment.

### 2.8 Weighted gene co-expression network analysis

After the genes with normalized FPKM value < 1 were filtered, the remaining genes were subjected to WGCNA. The genes with similar expression profiles were clustered into the same module, and the gene co-expression modules were constructed using the WGCNA v1.72 R package. Based on the expression levels of constituent genes and the module eigengenes (ME), biological modules were classified. The module classification parameters were set as follows: a minimum power value, 14; ME cutoff, 0.25; and a minimum module size threshold, 30 (33). The Pearson correlation coefficient was computed between each module and hypoxic treatment to identify modules significantly correlated with hypoxic stress response. The modules with a Pearson correlation coefficient of > 0.5 and a P-value of < 0.05 were defined as hypoxia-related. Further, functions of the genes in these modules were investigated through GO and KEGG enrichment analyses. The intra-module connectivity and inter-module correlation were calculated using WGCNA package to reveal gene interaction networks. Further, co-expression network of the top 30 genes (in terms of centrality degree) was constructed using Cytoscape v3.8.2 to visualize their interactions (34). The hypoxia tolerance-related hub genes or candidate genes were identified, based on their connectivity within the network.

### 2.9 Analysis of Bayesian networks

The gene regulatory network (GRN) of the modular gene set was plotted using the R package CBNplot, and hypoxia response -related genes were screened with the threshold of FDR< 0.05 based on Bayesian model by previously reported method (29, 30). The WGCNA module genes related to hypoxia were used to construct Bayesian network.

### 2.10 qRT-PCR analysis

Twelve DEGs were randomly selected for qRT-PCR in all groups to verify the accuracy of the sequencing results. Total RNA was extracted from the crucian carp gill tissue of hypoxic treatment groups (T1-T5, across 1 to 5 hypoxia) and the control group, as described in section 2.5. Primers were designed and synthesized by Sangon Biotech Co., Ltd. (Shanghai, China), which were listed in **Table 1**. The cDNA was synthesized according to the manufacturer’s protocol with Hiscript III Reverse Transcriptase kit (Vazyme, Nanjing, China). Quantitative real-time PCR (qRT-PCR) was performed using ChamQ Universal SYBR qPCR Master Mix kit (Vazyme, Nanjing, China). The 10 μL PCR reaction system contained 5.0 μL of the 2x SYBR qPCR Master Mix, 0.5 μL each of 10 μM forward and reverse primers (**Table 1**), 1 μL of cDNA template, and 3 μL of nuclease-free water. The qRT-PCR program was as follows: an initial denaturation at 95°C for 30 s, 40 cycles of 95°C for 5 s and 60°C for 30 s, followed by an extension at 95°C for 15 s, annealing at 60°C for 1 min, and final extension at 95°C for 15 s (35). Subsequently, a dissociation curve was obtained. The experiment was conducted with 3 replicates for each sample to ensure reproducibility, and 18s rRNA gene served as an internal control sample. The relative expression of gene was calculated using the 2^-ΔΔCt^ method (36).

**Table 1:** Specific primers for twelve verified genes used for qRT-PCR.

Primer name	Forward primer (5′–3′)	Reverse primer (5′–3′)
18S	ACCACATCCAAGGAAGGCAG	CACCAGACTTGCCCTCCAAT
mcm2	AGACAAGGTGGCCCGAATTT	GCCAGATACTGTGCAAACGTC
mcm6	GTCAATGGTGAAAGCGCCAA	CTTAAGCGCAGTCTCGTCCT
ripk3	GAAACGGCTGTGAGTTACGC	GTTTGCCGCTGTGTTGTTCT
caspase8	GTTGGAGAATCTGTGCCCCA	AGGCATCTGCTTGAAGACCG
hsp90	TCATCCGCAAGAACCTGGTC	TTGAGGGACACCATCTCGTC
znf395a	AGCTCAACCAGCACGTTCAT	AAAGCTTGAGTTGCACTCCC
caspase3	AAGAGGACGGCATGGTTGAG	TACCCTGGAGATGTGGCGTA
cdk2	TGCCAGACTACAAACCCTCC	CCGATGAACAAGGGCGTTCT
ccne	GTGAATGCCAGCAAGCAAGA	ACGAGGCGGATACAACTTCA
cdc23	AACCTGCTCTATGTGCGGAG	TCAGAGCCCGCTGGAAATAC
vegf	GTGGTGCCATTCATGGAGGT	ATGTGCTCGGTGTCATCAGG
cox7c	AGAGAACAAGTGGAGGCTTCT	GACAACAATGAATGGGAAGGTG

### 2.11 Statistical analysis

Statistical analysis of data was conducted using SPSS 22.0 software (SPSS Inc., Chicago, IL, USA). Normal distribution and variance homogeneity were assessed via the Shapiro-Wilk and Levene tests, respectively. Inter-group differences (hypoxia vs. control) were determined by independent samples t-tests, while intra-group differences were examined through one-way ANOVA. *P* < 0.05 was considered as statistically significant.

## 3 Results

### 3.1 Oxidative stress indexes of crucian carp gill under hypoxia stress

**Figure 1:**
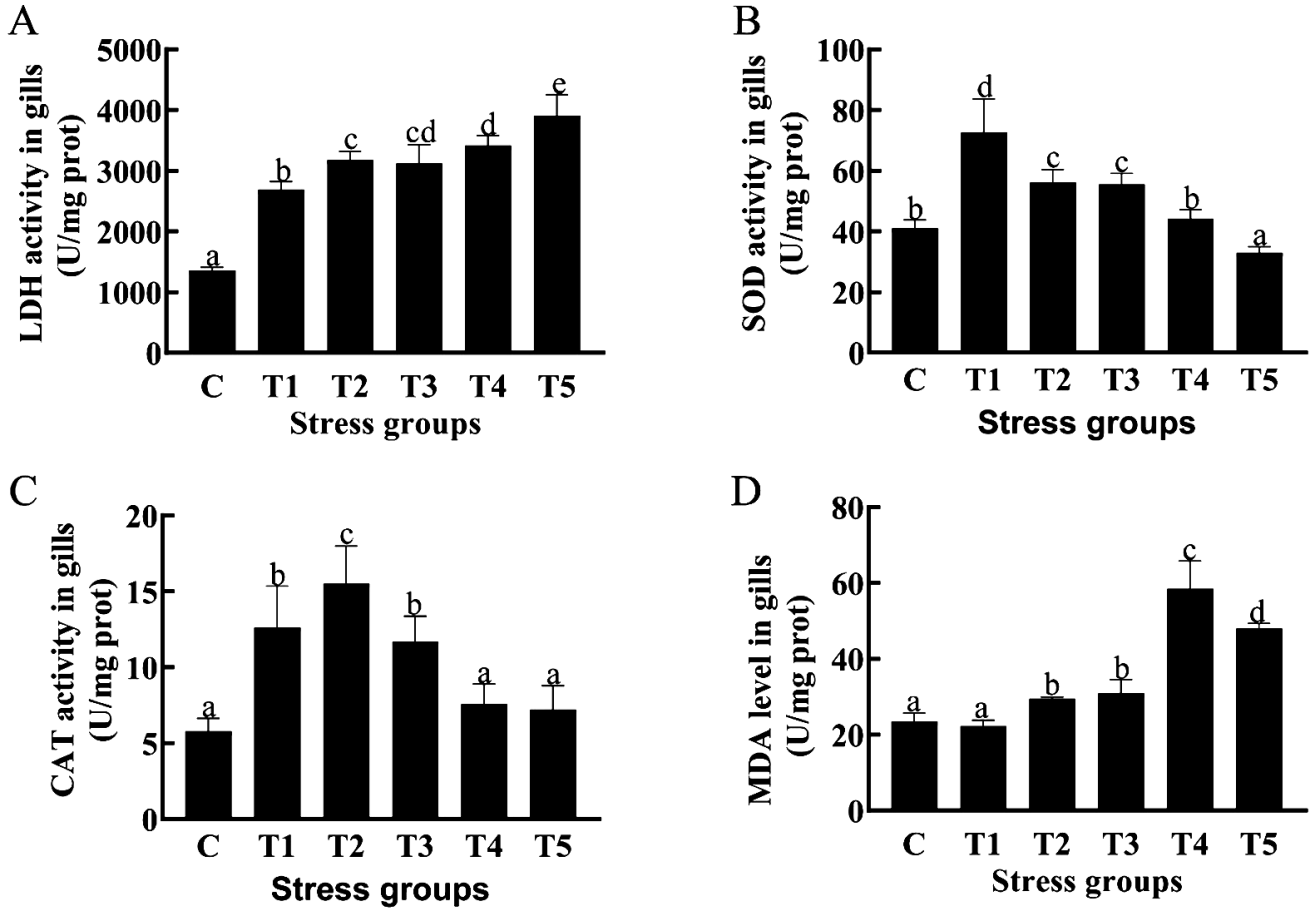
Effects of different durations of hypoxia stress on antioxidase activity and oxidation product MDA of Crucian carp gill. LDH activity in gills **(A)**;SOD activity in gills **(B)**; CAT activity in gills **(C)**; MDA level in gills **(D)**. Different letters above the bars indicate significant differences (p < 0.05).

We measured gill biochemical parameters of crucian carp at 24 h, 48 h,72 h,96 h and 120 h (T1-5) post hypoxia treatment (**Figure 1**). The results showed that the gill LDH activity was significantly higher (*P* < 0.05) in the all 5 hypoxia-treated groups (T1-T5) than in the control group (**Figure 1A**); the gill SOD activity was significantly higher inT1,T2, andT4 groups (*P* < 0.05) than in the control group (**Figure 1B**); the gill CAT activity was significantly higher (*P* < 0.05) in the 3 hypoxia-treated groups (T1-T3) than in the control group (**Figure 1C**); and gill MDA content was significantly higher (*P* < 0.05) in 3 hypoxia-treated groups (T3-T5) than in the control group (**Figure 1D**). Taken together, hypexia-treated groups exhibited a higher anti-oxidative enzyme activity after 3-day hypexia stress and a higher oxidation product content after 4-5 days of hypoxia stress than in control group.

### 3.2 Histological analysis of crucian carp gill under hypoxia stress

**Figure 2:**
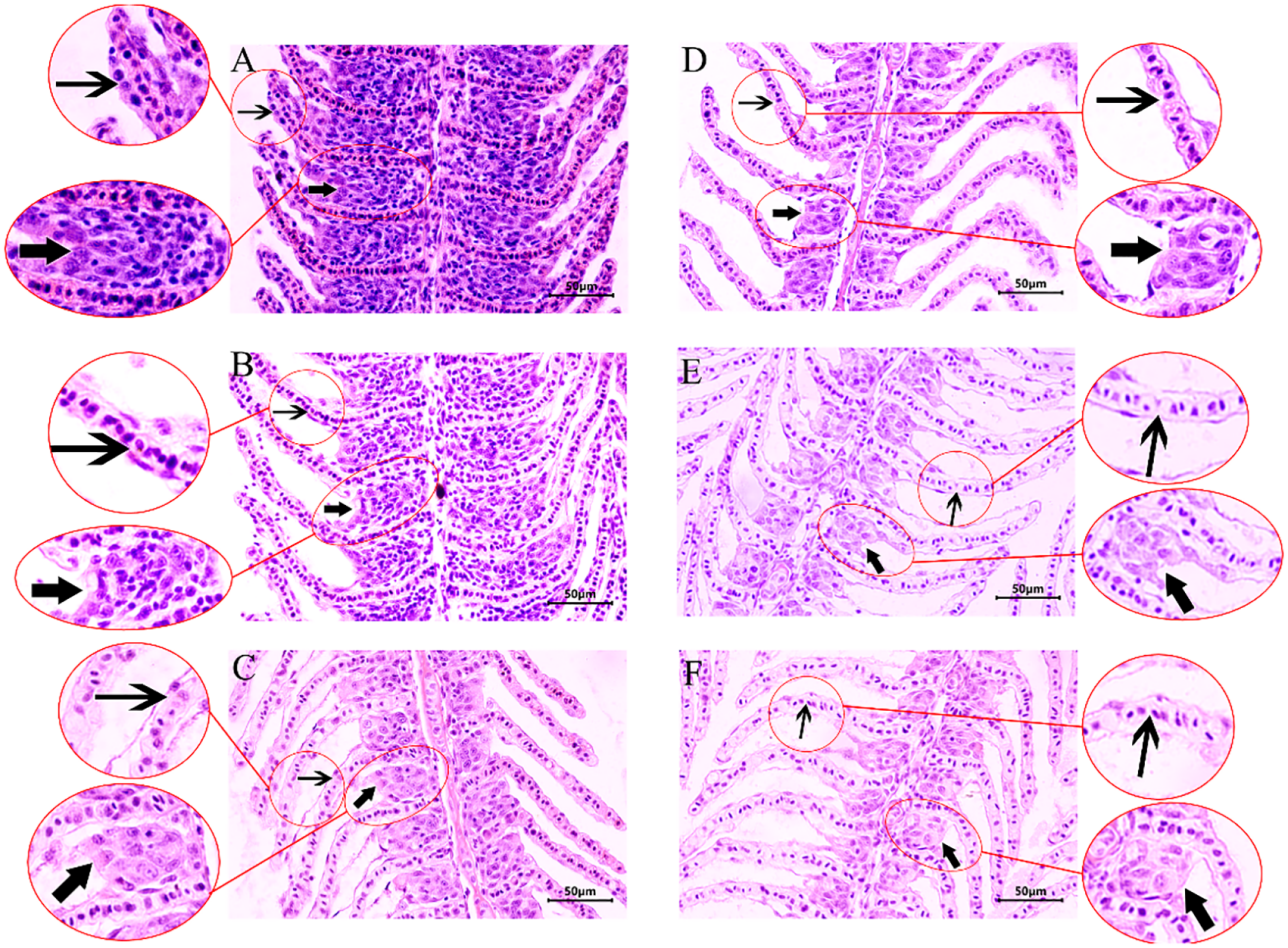
Hematoxylin–eosin (HE) staining of crucian crap gill tissues under hypoxic stress. **(A)** Structure of a normal gill. **(B–F)** Structure of gill exposed to hypoxia for 1, 2, 3, 4, 5 day(s). Thick arrow: ILCM; thin arrow: gill lamellae.

In the control group, crucian carp gill filament and lamella structural integrity and internal cell morphology were normal with neat arrangement (**Figure 2A**). In contrast, in the hypoxia treatment group, the cells surrounding the gill lamellae were gradually decreased; the gill interlamellar cell mass was reduced; and the epithelial cells in the gill lamellae became sparse with loose arrangement (**Figures 2B–F**).

### 3.3 Transcriptome sequencing and annotation

The gill tissues from fish with or without exposure to hypoxia were subjected to transcriptome sequencing on the L230 Illumina NovaSeq 6000 platform. After low-quality sequence removal, the number of clean reads in each of 18 sample libraries ranged from 41410340 to 48353576. The Q20 values of these 18 sequenced libraries ranged from 98.48% to 99.16%, whereas their GC contents ranged from 41.61% to 46.64%. These results confirmed the reliability of the transcriptome sequencing data. The transcriptome sequencing data of control group and hypoxia-treated group were aligned to the crucian carp genome, respectively.

### 3.4 Identification of DEGs in the comparison of hypoxia groups vs. control group

**Figure 3:**
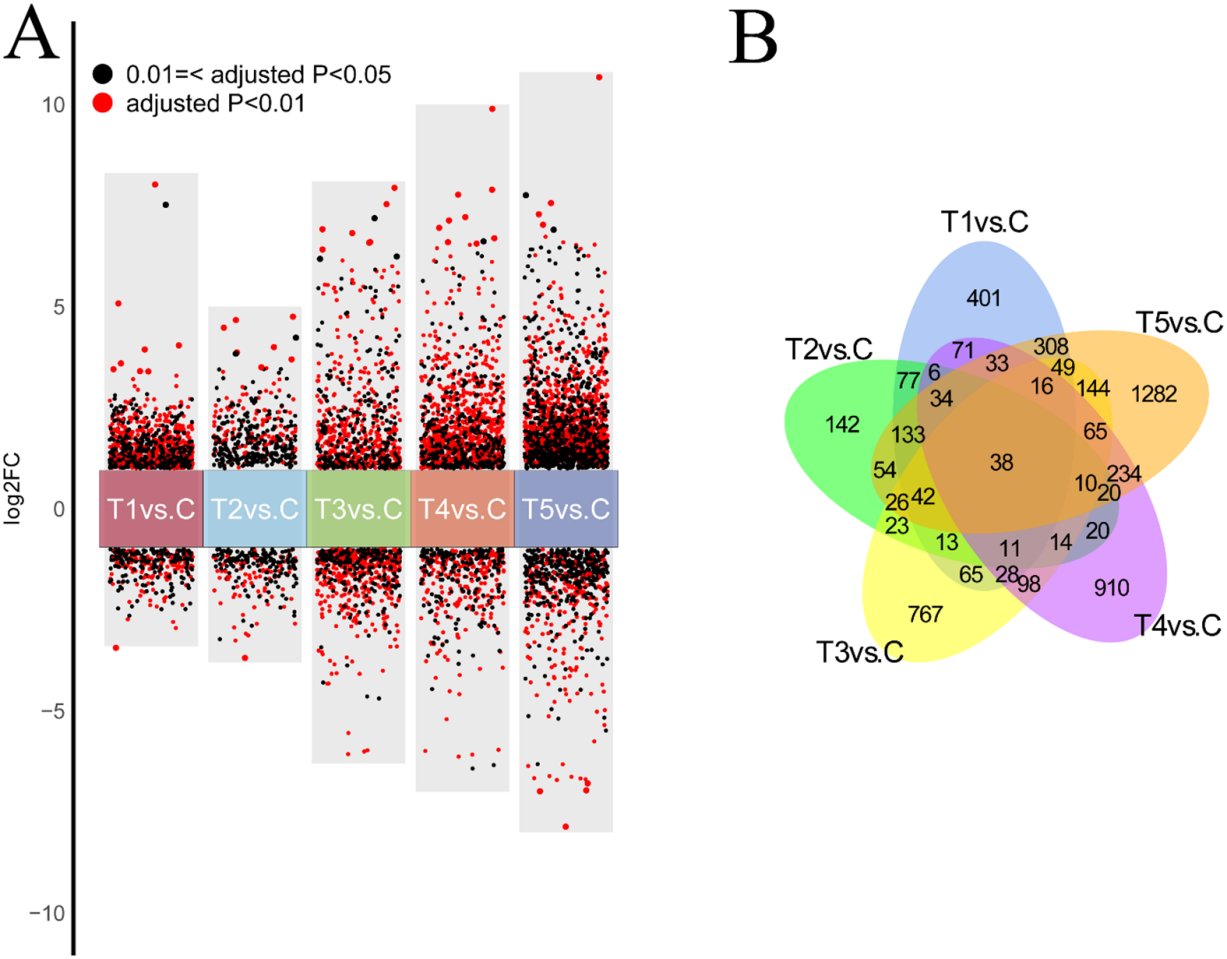
**(A)** Volcano plot of DEGs in crucian crap gills under hypoxic stress in the comparison of hypoxia-treated groups vs. control group. Log_2_ FC <-1 indicates down-regulated DEGs; Log_2_ FC >1 represents up-regulated DEGs. 2. **(B)** Venn plot of DEGs in crucian crap gills under hypoxic stress in the comparison of hypoxia-treated groups vs. control group.

The DEGs in the hypoxia treatment vs. control groups were identified on the basis of |log2 FC| ≥ 1 and P < 0.01 (**Figure 3A**). A total of 3,502 DEGs were identified across five comparison groups. The DEGs were identified in each of 5 comparisons (T1, T2, T3, T4, or T5 vs. C) and the largest number of DEGs (1,282) were identified in the comparison of T5 vs. C, accounting for 36.60% (**Figure 3A**). Venn diagrams revealed that DEGs were shared by the five comparation groups, and 401, 142, 767, 910, and 1,282 DEGs were unique to the comparison group of T1 vs. C, T2 vs. C, T3 vs. C, T4 vs. C, and T5 vs. C, respectively (**Figure 3B**).

### 3.5 GO and KEGG analyses of DEGs

**Figure 4:**
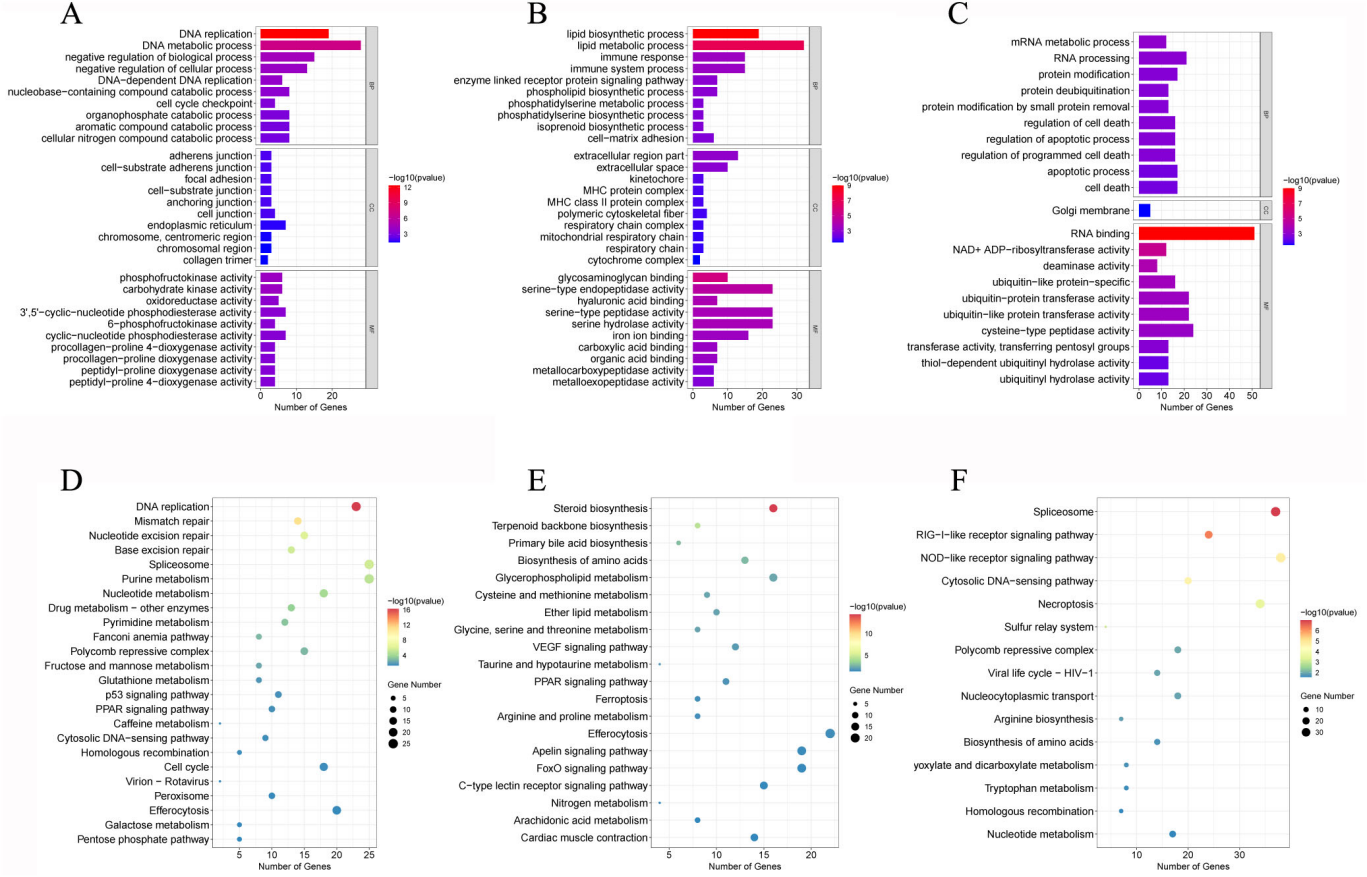
GO enrichment analysis of DEGs in T1 vs. C **(A)**, T3 vs. C **(B)**, and T5 vs. C **(C)**. KEGG pathway enrichment analysis of DEGs in T1 vs. C **(D)**, T3 vs. C **(E)**, and T5 vs. C **(F)**.

In the GO enrichment analysis, the DEGs were assigned to 123 GO terms, including 35 biological process (BP), 30 cellular component (CC), and 40 molecular function (MF) (**Figure 4**, **Supplementary Figure S1**). The top GO terms were DNA replication (**Figure 4A**) and lipid biosynthetic process for BP (**Figure 4B**), and it was RNA binding for MF (**Figure 4C**). These results indicated substantial changes in immune defenses and energy metabolism in gill tissues under hypoxic stress.

In the KEGG enrichment analysis, 18 hypoxia-related pathways in crucian carp gill post 5-day hypoxic stress were significantly enriched, mainly including cell cycle, nucleotide metabolism, p53 signaling pathway, glutathione metabolism (**Figure 4D**), drug metabolism - other enzymes, NOD-like receptor signaling pathway (**Figure 4E**), PPAR signaling pathway, VEGF signaling pathway (**Figure 4F**, **Supplementary Figure S2**).

### 3.6 Module analysis based on WGCNA

**Figure 5:**
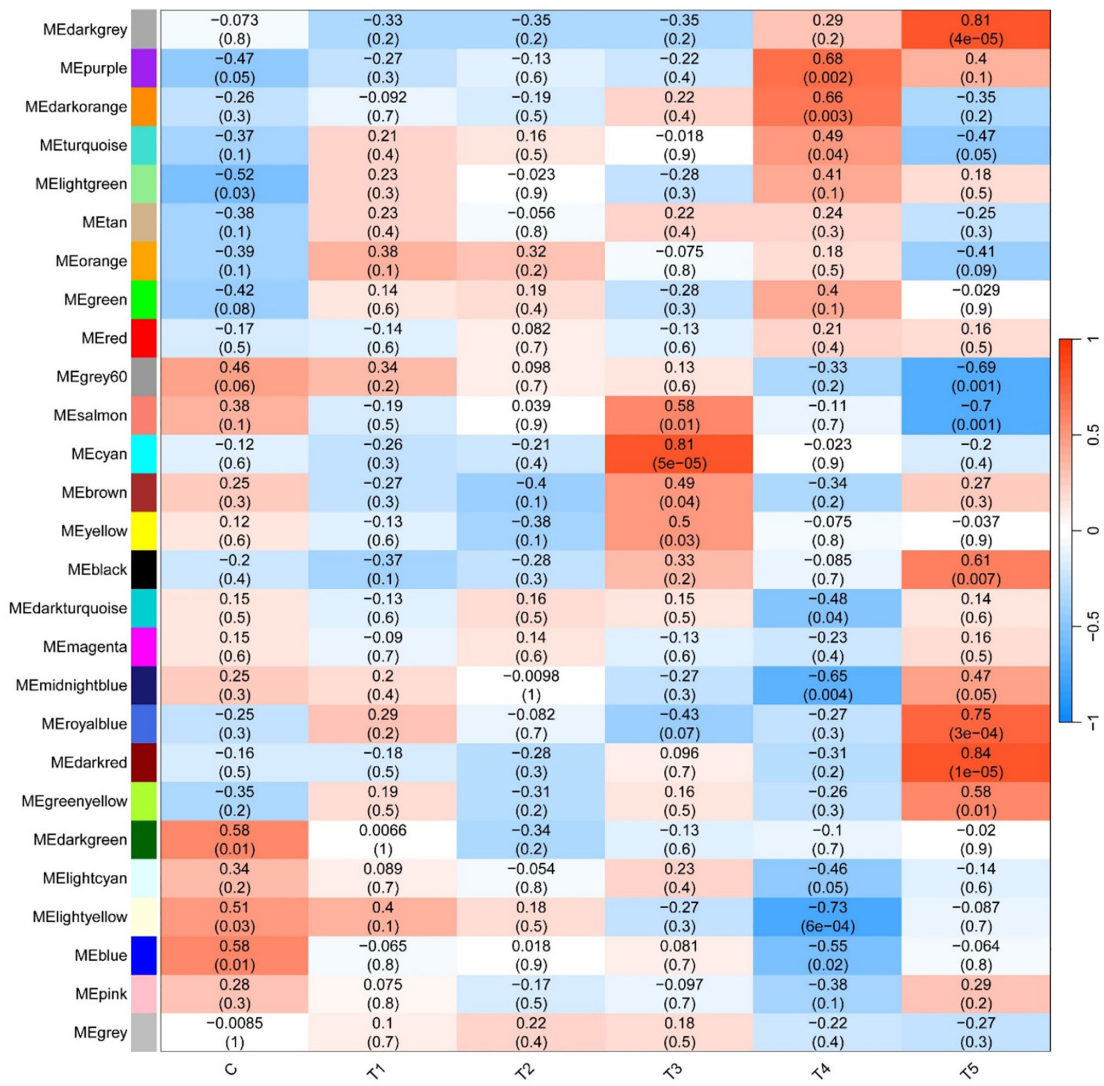
Correlation heatmap between modules and hypoxia treatments. The color scale on the right shows module-hypoxia treatment correlation coefficients from - 1 (blue) to 1 (red). The numbers in brackets represent significance (P < 0.05).

**Figure 6:**
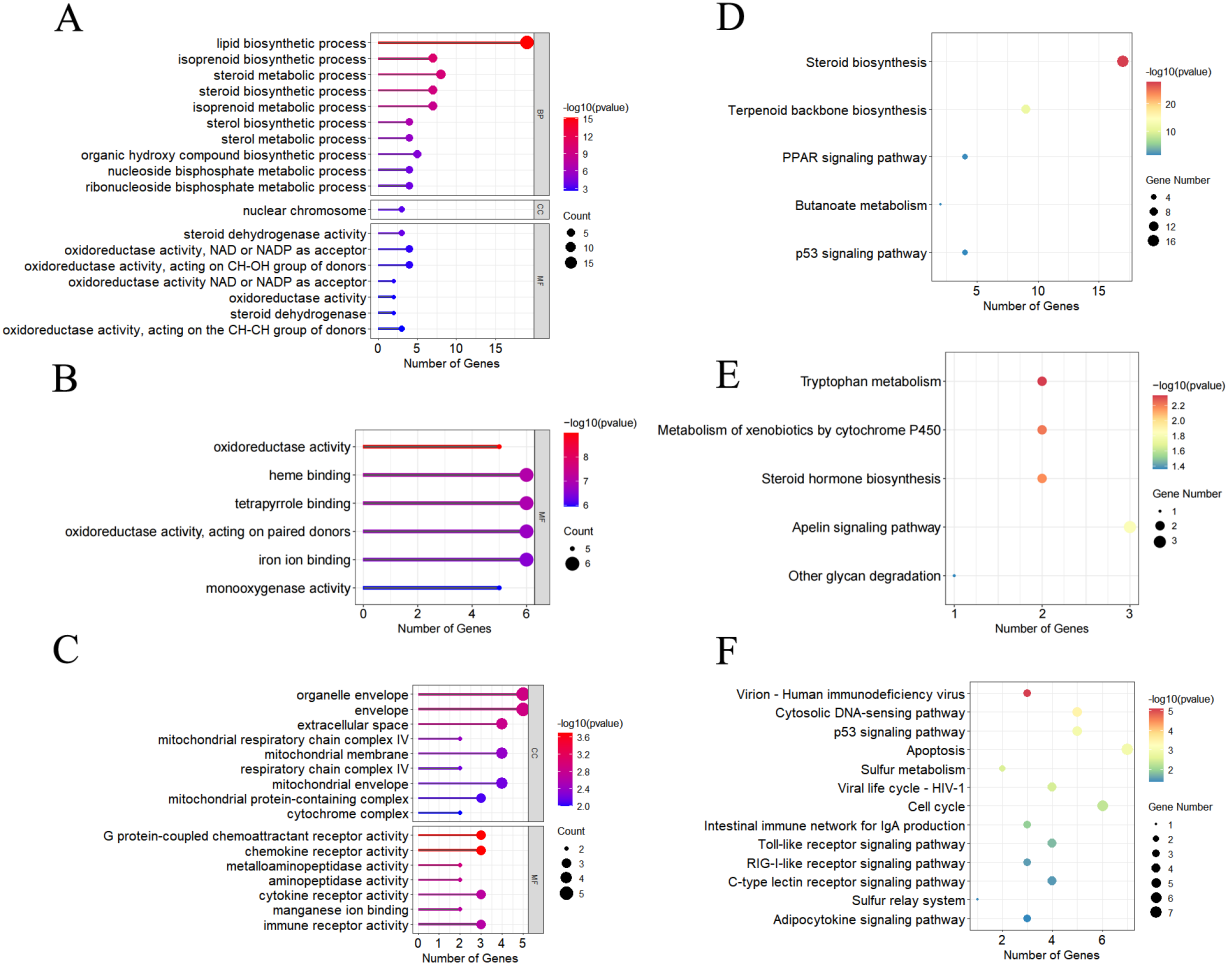
Significantly enriched GO terms by GO enrichment analysis in MEcyan module **(A)**, MEdarkgrey module **(B)**, and MEdarkred module **(C)**. Significantly enriched pathways by the KEGG enrichment analysis in MEcyan module **(D)**, MEdarkgrey module **(E)**, and MEdarkred module **(F)**, respectively.

By WGCNA, a total of 27 modules under hypoxia stress were identified. As shown in **Figure 5**, the MEcyan module (R = 0.81, p = 5e-05), the ME darkgrey module (R = 0.81, p = 4e-05), and the MEdarked module (R = 0.84, p = 1e-05) were significantly correlated with T3 and T5, respectively. GO enrichment analysis revealed that lipid biosynthetic process, oxidoreductase activity, and organelle envelope were significantly enriched GO terms in cyan, darkgrey, darked modules, respectively (**Figures 6A–C**). KEGG enrichment analysis found that steriod biosynthesis, PPAR signaling pathway, and p53 signaling pathway were significantly enriched in cyan module; tryptophan metabolism, cyp450 metabolism, and apelin signaling pathway in darkgrey module; RIG-I-like receptor signaling pathway, toll-like receptor signaling pathway, and apoptosis in darked module (**Figures 6D–F**).

**Figure 7:**
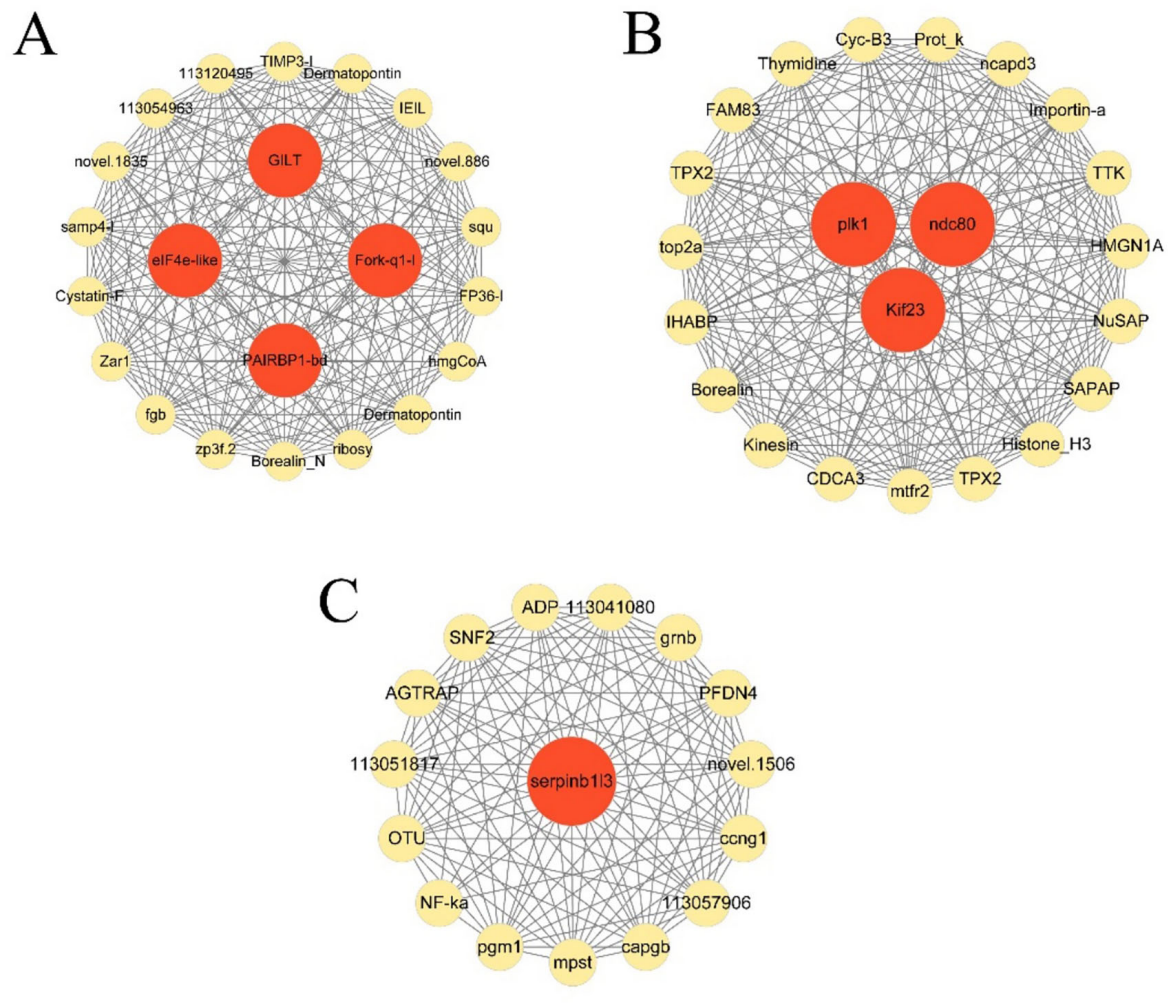
Gene co-expression networks of top 22 DEGs in cyan module **(A)**, top16 genes in darkgrey module **(B)**, and top 23 genes in darked module **(C)**.

The hub genes in each module and connectivity with other genes were further explored by the WGCNA. As illustrated in **Figure 7**, there were 26 genes and 4 hub genes (*elF4e-L*, *Fork-q1-l*, *PAIRBP*, and *GILT*), 16 genes and 1 hub genes (*serpinb1/3*) and 22 genes and 3 hub genes (*ndc80*, *plk1* and *Kif23*) in the cyan module, darked module, and darkgrey module, respectively. These 8 hub genes in 3 modules exhibited high connectivity with other genes, indicating that they played pivotal roles in responses to hypoxia stress in these 3 modules.

### 3.7 Bayesian network graph of DEGs

**Figure 8:**
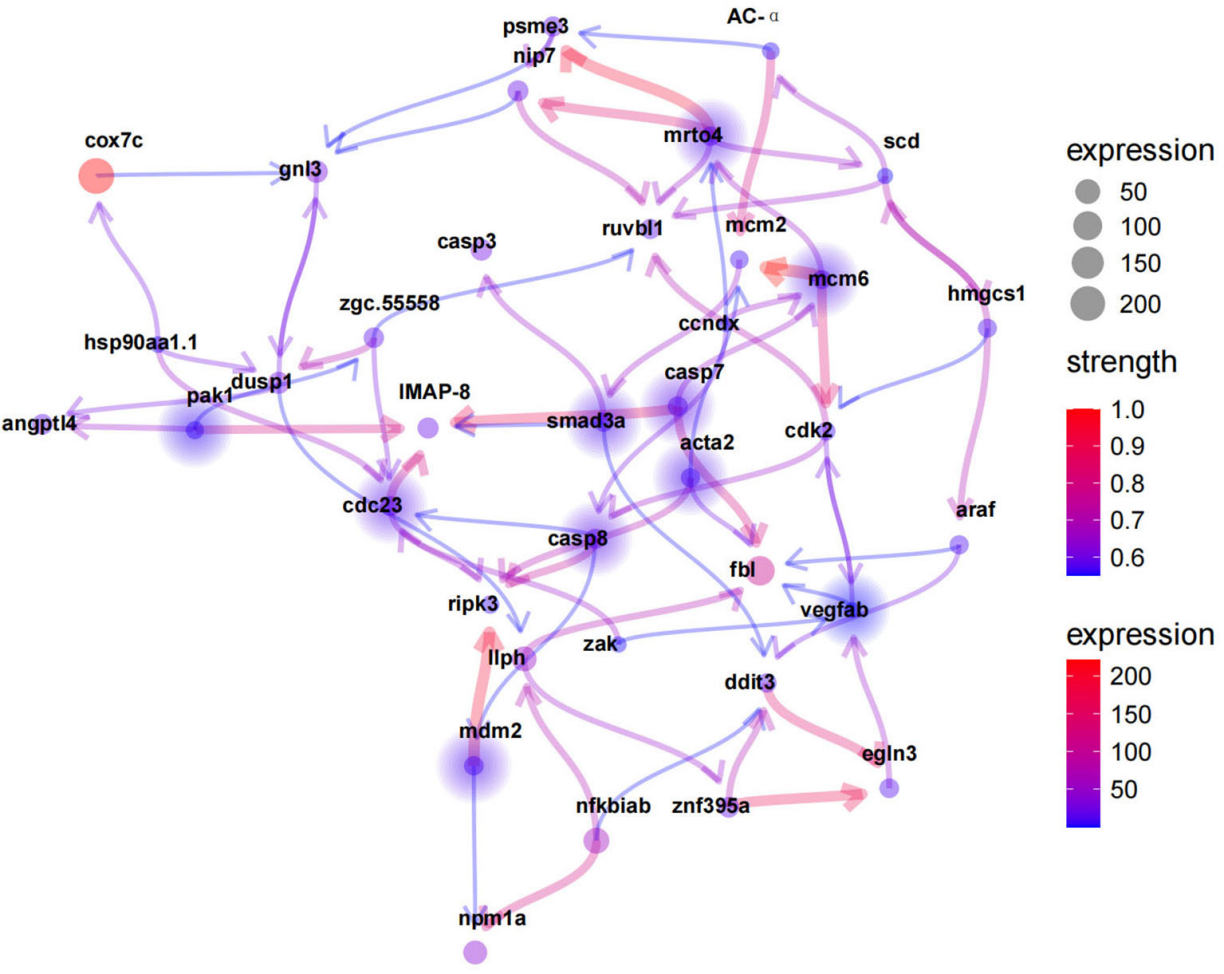
Hypoxia-relatedkey genes predicted by Bayesian network analysis. Solid circles indicate key genes. Line color from blue to red represent the increasing strength.

Eleven hypoxia-related key genes were screened from the above mentioned 3 hypoxia modules with a threshold of p value < 0.05, including *pak1, cdc23, smad3a, caspase7, caspase8, mdm2, mcm6, acta2, egln3, vegf*, and *mrto4*. The Bayesian network graph of DEGs was plotted using the R package CBNplot (https://noriakis.github.io/software/CBNplot/) (**Figure 8**).

### 3.8 Validation of gene expression by qRT-PCR

**Figure 9:**
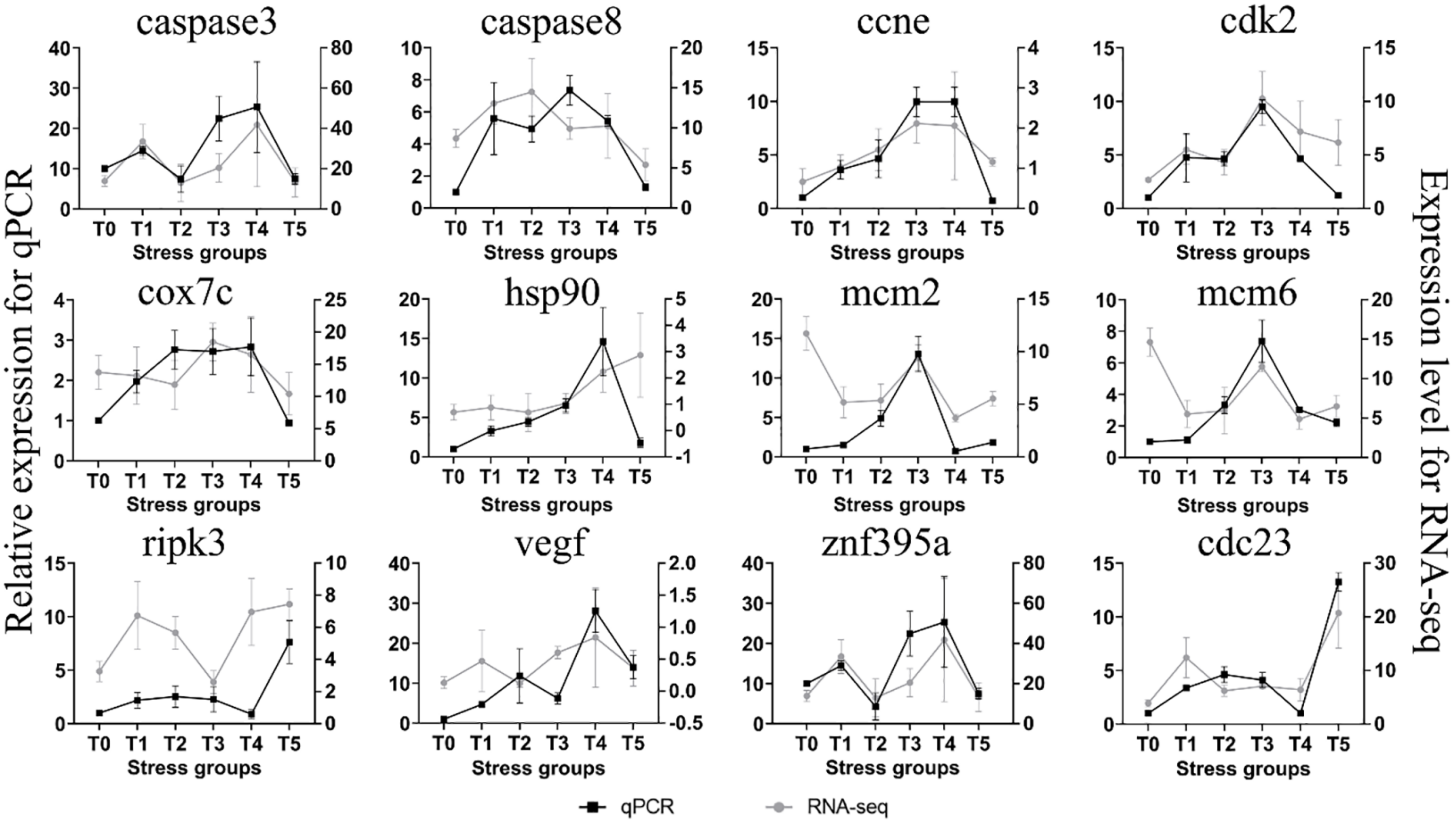
qRT-PCR analysis of 12 randomly selected genes for the validation of RNA-Seq data.

To verify the accuracy of the RNA-Seq data, we selected 12 genes for qRT-PCR. The results of qRT-PCR (**Figure 9**) indicated that the expression patterns (upregulation and downregulation) of 6 genes, including *caspase8, cnne, caspase3, znf395a, cdk2*, and *cdc23*, were consistent with the change trends of the transcriptome data (FPKM). The correlation analysis showed that the levels of the measured gene transcripts had a high linear correlation (R² = 0.81), indicating that the transcriptome sequencing data were reliable.

## 4 Discussion

### 4.1 Effects of hypoxia stress on histology and antioxidative capacity of crucian carp gill

Our study found that hypoxia leads to a reduction in the volume and thickness of the interlamellar cell masses in crucian carp. This represents a morphological change in the gills of fish in response to environmental changes, which reduces the gas diffusion distance between blood and water. Consequently, it exposes the gill lamellae responsible for respiratory functions (37). It has been reported that many farmed teleost fish, such as grass carp (38) and white bream (39), also possess this ability. However, the species *M. Pellegrini* has been shown not to exhibit this capability under hypoxic conditions (40). As the duration of hypoxia increases, the changes in the interlamellar cell masses become more pronounced, increasing the respiratory surface area of the fish gills. This enhancement facilitates oxygen absorption from the water and aids fish in surviving in environments with low dissolved oxygen (11). The present study also found that the reduction of interlamellar cell masses can bring some harm to fish, including changes in osmotic pressure that increase energy demands, greater exposure to toxic substances and parasites, and a higher risk of mechanical damage to the gills (38). Therefore, when there is sufficient dissolved oxygen in the water, it is prudent for fish to maintain a smaller gill surface area.

Hypoxia has been reported to lead to an imbalance between oxidation and antioxidation in organisms (41), which induces oxidative stress and excessive ROS in various fish species (42, 43). The antioxidant system in fish includes multiple antioxidative enzymes such as LDH, SOD, and CAT (44). Under hypoxic stress, enhancing antioxidant capacity can prevent oxidative damage in fish and eliminate excessive ROS (45). Our data showed that SOD and CAT levels were significantly higher in the hypoxia treatment groups than in the control group under the first 3-day hypoxic stress, indicating an increase in antioxidant capacity, but a decrease was observed in the last two days (day 4-5) of the hypoxia experiment. This might be due to the damage to the gill lamellae induced by prolonged exposure to hypoxia, which was supported by vacuole formation in the gill lamellae was observed on day 4 and day 5. LDH is a key enzyme in anaerobic metabolism, and it can reversibly catalyze the production of pyruvate from lactate (46). It has been reported that hypoxic stress can increase LDH activity in the gills of Nile tilapia (*O. niloticus*), thus enhancing its anaerobic metabolic capacity. In addition, the increased LDH activity has also been detected in the gills of spotted fish (*Oplegnathus punctatus*) under hypoxic conditions (47). In this study, LDH activity was consistently significantly elevated in various hypoxia groups. Based on the above findings, we speculated that at various stages of hypoxic stress, crucian carp might increase LDH enzyme activity, thus enhancing anaerobic metabolism and maintaining energy supply. The upregulation of *LDH* gene can catalyze the conversion of lactate to pyruvate to participate in anaerobic glycolysis, and thus the expression level of *LDH* gene can reflect the level of anaerobic glycolysis to some extent (12).

### 4.2 Effects of hypoxia on signal transduction

Cells possess highly complex interactive networks that respond specifically to various stimuli. When cells encounter hypoxic stress, multiple cellular signaling pathways are activated to maintain oxygen homeostasis, and cells adjust their physiological functions to respond to hypoxic stress (48). In rainbow trout (49), yellow catfish (*Tachysurus fulvidraco* ♀×*Pseudobagrus vachellii* ♂) (1), and dark barbel catfish (*Pelteobagrus vachelli*) (50), signal transduction pathways are activated during hypoxic stimulation. Our data showed that under hypoxic stress, p53 signaling pathway was activated in crucian carp gill, thus enhancing anaerobic metabolism through glycolysis and increasing energy supply through lipid metabolism, eventually maintaining cellular homeostasis. Moreover, under hypoxic stress, mitochondrial respiration is inhibited, releasing signaling molecule cyp450 into the cytoplasm to activate apoptosis genes of the caspase family, leading to extrinsic apoptosis in carp fish (51). Long-term hypoxia leads to the production of inflammatory factors, thus the activating immune signaling pathways such as RIG-I and NLRs in cells to eliminate these inflammatory factors. These findings suggested that in crucian carp, cellular apoptotic pathways might be initially triggered under hypoxic stress, and prolonged exposure to hypoxia might further induce the activation of immune pathways associated with endogenous antioxidant defense mechanisms.

### 4.3 Effect of hypoxia stress on apoptosis-related genes of crucian carp gill

*Hif-1α*, as a primary regulator of the hypoxic response, can regulate a series of adaptive responses to hypoxic stress (52). In this study, the *hif-1α* gene was consistently lowly expressed of hypoxic stress, but its downstream target gene *vegf* was significantly up-regulated. The upregulation of the *vegf* gene under hypoxic conditions may enhance angiogenesis, which is crucial for gill remodeling (53). Our data also showed that the expression of *egln3* gene (the hydroxylase of *hif-1α*) was significantly increased. *Egln3*can promote the hydroxylation of *hif-1α*, leading to its ubiquitination and subsequent degradation by the proteasome (54). This might explain its negligible low expression of the *hif-1α*under hypoxic stress. In addition, we have identified 11 key regulatory genes, and these genes might play a crucial role in the response of crucian carp gill to hypoxia. In the initial stages (day 1 and day 2) of hypoxia experiment, a significant accumulation of ROS resulted in the up-regulation of cytochrome P450 (*cyp450*), thus triggering the up-regulationof*caspase3/8* apoptosis-related genes. Key genes in the cell cycle such as *cdk2*, *mcm6*, and *prmt1* play significant roles in cell apoptosis. Gene *Smad3a* can down-regulate *cdk2*, hindering cell proliferation in the G1 phase, and gene*prmt1* acts on the Fox1 receptor to release signaling factors into the cytoplasm, and these signaling factors act on apoptosis-related genes *caspase8* and *caspase7*. Under severe hypoxic conditions, the expression of the *ripk3* gene triggers necroptosis. In previous study, under hypoxia stress, *hif-1α*activates the VEGF pathway, thereby inducing angiogenesis (55). The *vegf* gene is an important gene promoting angiogenesis, and accordingly, heterozygous *vegf* mutant mice embryo die *in utero* due to vascular defects (52). In this study, the activation of the VEGF pathway might also be related to Pak1 enzyme. Recently, Kelly et al. (56) have reported that *Gata6* gene in the Pak1/Erk signaling module regulates cardiovascular development in zebrafish. The expression of the *vegf* gene contributes to the uptake of oxygen in blood, thus facilitating adaptation to hypoxic conditions. Our data showed that hypoxia significantly reduced ILCM between crucian carp gill lamellae, thus increasing the contact area between gill lamellae and water and accelerating oxygen intake by gill (57). Therefore, we speculated that high adaptability of crucian carp to hypoxic conditions might be closely related to the apoptosis of ILCM and the action of the *vegf* gene.

## 5 Conclusion

In this study, our transcriptome analysis revealed that the differentially expressed genes in the gills of crucian carp under hypoxic conditions are mostly related to energy metabolism, antioxidant stress, and apoptosis pathways. In the first two days of the hypoxia experiment, crucian carp reduces oxidative damage caused by reactive oxygen species (ROS) through anaerobic metabolism and activates pathways such as P53 to block the cell cycle and affect cell proliferation, including genes like *pak1* and *cdk2*, which are important for promoting apoptosis in interlamellar cells (ILCM) and help crucian carp to improve oxygen uptake efficiency in the early stages of hypoxia, thus adapting to low oxygen conditions. Secondly, there is the intrinsic apoptotic pathway dominated by the caspase family. However, under prolonged hypoxia, the gills of crucian carp will strengthen their adaptation to hypoxia by generating microvessels, such as *vegf*. In the later stages of hypoxia, it may be due to cell necrosis causing a large number of inflammatory factors, leading to the enrichment and activation of cellular immune pathways such as RIG-I and NLR. While the transcriptome analysis provided valuable insights into gene expression changes, it lacked in-depth functional validation of the identified genes. Merely observing altered expression levels does not definitively prove their direct contribution to hypoxia adaptation, and further experimental confirmation through techniques like gene knockout or overexpression need to be conducted to ascertain their true functional significance.

## Data Availability

The datasets presented in this study can be found in online repositories. The names of the repository/repositories and accession number(s) can be found in the article/**Supplementary Material**.
