# Supplementary material for: Transcriptome analysis reveals hypoxic response key genes and modules as well as adaptive mechanism of crucian carp (Carassius auratus) gill under hypoxic stress
Source: Front Immunol. 2025 Feb 5;16:1543605. doi: 10.3389/fimmu.2025.1543605 (PMC11835930; doi:10.3389/fimmu.2025.1543605)
Supplement: Supplementary file 1 [file DataSheet1.docx]

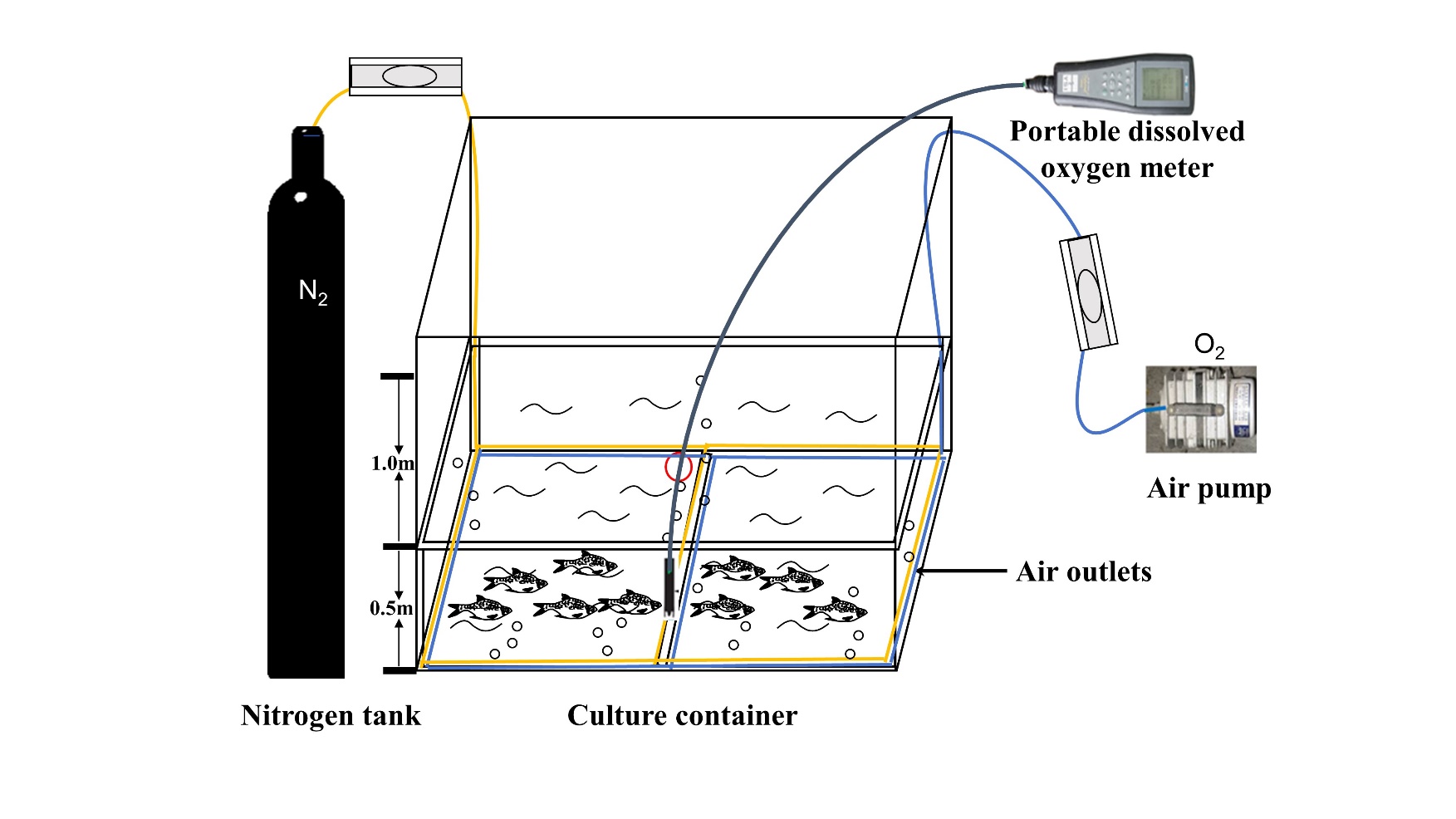


**Supplementary Fig. 1.** Schematic of hypoxia-tolerant crucian carp breeding under hypoxic condition (DO concentration = 0.6 ± 0.3mg·L^–1^). The water depth in the glass tank was 1.5 m, and a100-mesh sieve covered by glass was placed 1 m below the water surface to prevent the fish from accessing the air-water interface in the tank. Nitrogen (N_2_) and air were introduced into glass tank via a gas disc in order to decrease the DO concentration, and the flow rate of introduced gas was adjusted through the gas flow regulator to maintain low DO concentration (DO= 0.6 ± 0.3mg·L^–1^). The DO concentration in the glass tank was measured with DO meter.


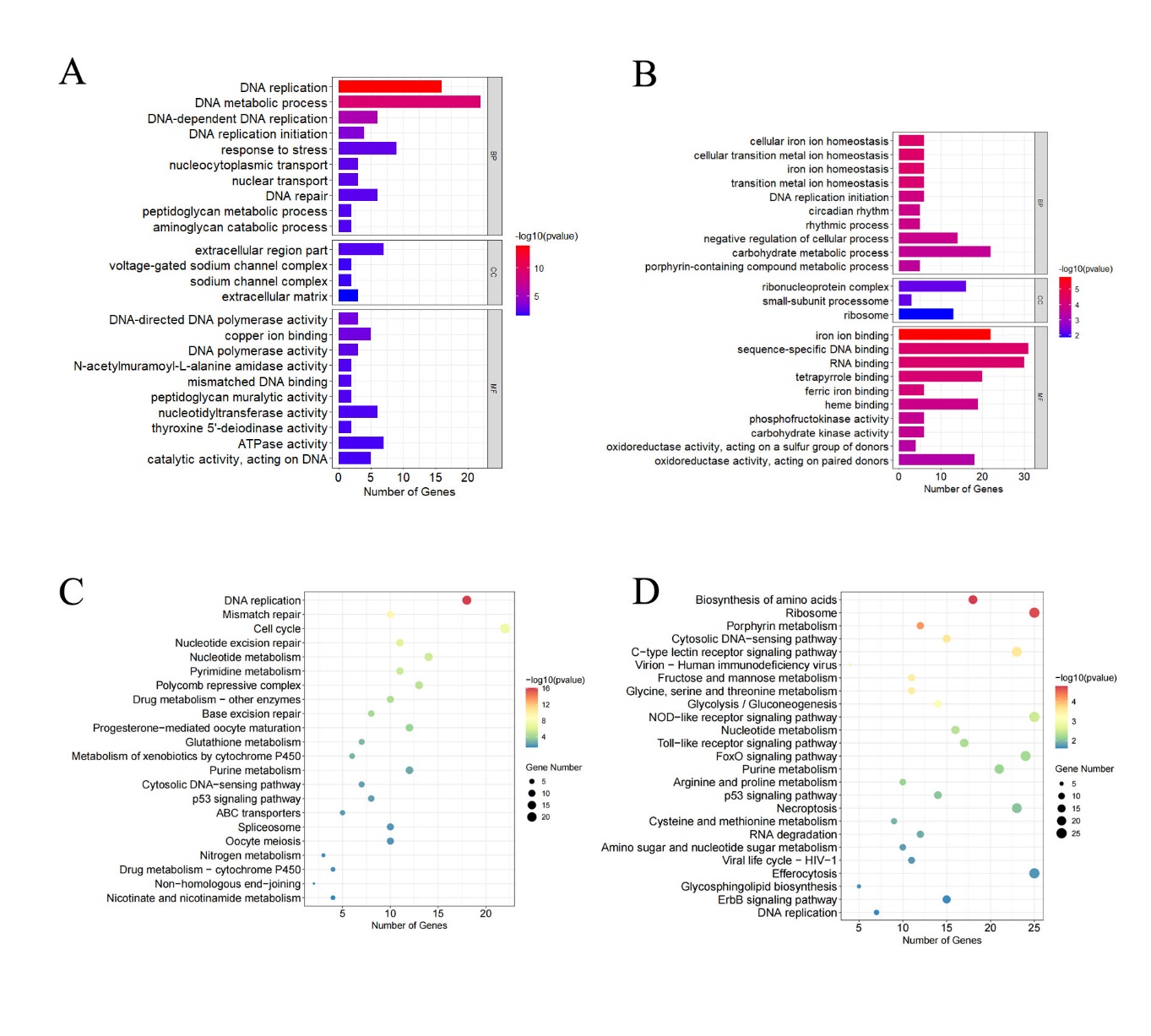


**Supplementary Fig. 2.** (A) GO enrichment analysis of DEGs in T2 vs. C and (B) T4 vs. C. KEGG enrichment analysis of DEGs in (C) T2 vs. C and (D) T4 vs. C.
